# Bridging Disciplines to Form a New One: The Emergence of Forensic Genetic Genealogy

**DOI:** 10.3390/genes13081381

**Published:** 2022-08-01

**Authors:** Claire L. Glynn

**Affiliations:** Department of Forensic Science, Henry C. Lee College of Criminal Justice and Forensic Sciences, University of New Haven, West Haven, CT 06516, USA; cglynn@newhaven.edu

**Keywords:** Forensic Genetic Genealogy, forensic genetics, DNA, Single Nucleotide Polymorphisms, forensic science, investigations, consumer DNA testing, genealogy

## Abstract

Forensic Genetic Genealogy (FGG) has fast become a popular tool in criminal investigations since it first emerged in 2018. FGG is a novel investigatory tool that has been applied to hundreds of unresolved cold cases in the United States to generate investigative leads and identify unknown individuals. Consumer DNA testing and the public’s increased curiosity about their own DNA and genetic ancestry, have greatly contributed to the availability of human genetic data. Genetic genealogy has been a field of study/interest for many years as both amateur and professional genetic genealogists use consumer DNA data to explore genetic connections in family trees. FGG encompasses this knowledge by applying advanced sequencing technologies to forensic DNA evidence samples and by performing genetic genealogy methods and genealogical research, to produce possible identities of unknown perpetrators of violent crimes and unidentified human remains. This combination of forensic genetics, genetic genealogy, and genealogical research has formed a new subdiscipline within the forensic sciences. This paper will summarize the individual disciplines that led to the emergence of FGG, its practice in forensic investigations, and current/future considerations for its use.

## 1. Introduction

Forensic Genetic Genealogy (FGG), also known as Investigative Genetic Genealogy (IGG), emerged as a novel investigative tool in 2018, and combines the fields of forensic genetics with both genetic and conventional genealogy. With the rise in popularity of consumer DNA testing there now exist public genetic genealogy databases populated with large amounts of genetic data from consumers, i.e., general members of the public. FGG utilizes this mass of data for the purposes of human identification in a forensic context. FGG expands the field of forensic genetics and does not replace forensic DNA profiling, instead it complements it. The use of FGG to resolve the identity of the Golden State Killer in 2018 is widely credited as the case that brought FGG to the forefront, spurring widespread interest from the forensic/law enforcement communities and the general public. Since then, the use of FGG in forensic/criminal investigations has experienced rapid growth within the United States (US). FGG is predominantly used in investigations to identify perpetrators of violent crimes (e.g., homicide and sexual assault) and to identify unidentified human remains (UHRs), commonly known in the public as Jane and John Doe cases. It is estimated that over five hundred cases have benefited from the use of FGG, with no exact data available as there is currently no required reporting of its use. The majority of cases solved using FGG have been in the US, however, some countries, such as Sweden [1], have reported the successful use of FGG in their forensic casework, while other countries, such as the United Kingdom (UK) [2] and Australia [3], are considering it for potential future use.

FGG should not be confused with Forensic Genealogy, which has long been in existence and typically uses non-DNA genealogical methods, albeit DNA evidence can sometimes be used to confirm conclusions. Forensic Genealogy typically refers to estate and probate cases to identify/find heirs, the identification of living descendants of fallen soldiers for their repatriation, and other historical investigations. FGG should also not be confused with Familial DNA Searching (FDS), which is an additional tool performed with DNA profiles that have been generated using traditional forensic DNA profiling. FDS has been in use since 2002, first in the UK [4,5], then later in other countries [6,7], and is limited to only some states in the US [8]. FDS is a deliberate search of a criminal DNA database to search for partial matches between forensic profiles and offender profiles in the database. The goal is to identify and statistically rank database profiles that are not a full match, but that share sufficient genetic similarity to suggest a possible close/immediate familial relationship, i.e., full sibling, parent–child, with limited accuracy at more distant levels. FGG involves searching genetic genealogy databases for individuals that share segments of matching, or very similar, DNA with an unknown DNA sample and are therefore considered genetically related at some level. The DNA matches/relatives identified in genetic genealogy databases are typically distant relatives, i.e., first cousins and beyond. Using genetic genealogy tools and genealogical research methods, e.g., record searching, family trees are constructed with the goal of identifying potential candidates(s) as the identity of the unknown DNA sample. It is important to note, at the time of writing, the only genetic genealogy databases that explicitly allow their sites to be used by law enforcement for FGG purposes are GEDmatch PRO, FamilyTreeDNA (Houston, TX, USA), and DNASolves, which will be discussed in a further section. This paper will discuss the factors and disciplines that led to the emergence of FGG in forensic investigations, the current status of FGG in the US, and the considerations for the future.

## 2. Forensic DNA Profiling and Forensic Genetic Genealogy

FGG differs from traditional forensic DNA profiling in many ways, most notably in the types of DNA markers analyzed, the technology used, the data generated, and the DNA databases searched, see Table 1. 

In Forensic DNA profiling, between 16 and 27 Short Tandem Repeat (STR) markers are analyzed using PCR amplification and capillary electrophoresis to generate an STR/DNA profile of a questioned/unknown sample, e.g., a biological sample deposited by an unknown perpetrator of a crime. The unknown STR profile can then be compared to known reference STR profiles, e.g., collected from known suspects in an investigation, and/or searched in a national DNA database. These DNA databases are typically composed of STR profiles from convicted offenders, arrestees, and other unknown forensic profiles collected from crime scenes. The number of STR markers analyzed are dependent on the individual country, with the US employing the Combined DNA Index System (CODIS) Core 20 STRs, the UK and Ireland employing DNA-17, etc. The individuals included in national DNA databases are also dependent on the laws and policies of individual countries and individual states in the US.

FGG does not use STR data, nor does it search national DNA databases. Instead, between ~600,000 and ~1 million Single Nucleotide Polymorphisms (SNPs) markers are analyzed in an unknown/questioned sample. The resulting SNP data is uploaded to genetic genealogy databases, which are populated with general members of the public who have voluntarily provided their DNA for SNP analysis or uploaded their SNP DNA data, i.e., GEDmatch, FamilyTreeDNA, and DNASolves. The unknown SNP data uploaded by law enforcement/FGG providers are compared to other users’ SNP data within the database(s) and a list of users who share some DNA with the unknown, and are therefore in some way genetically related, is generated. This list of genetic relatives, more commonly known as DNA matches, can range from close (e.g., sibling, parent–child) to distant (first cousins and beyond) relatives. The amount of DNA shared can then be used to compute probabilities for possible relationships to place DNA matches into predicted positions in the unknown individual’s family tree. Following this, documentary evidence, e.g., civil registration records (birth, marriage, death, etc.), plus other investigative evidence, can be used to build out the family trees of identified/verified DNA matches, typically beginning with identifying who the DNA match’s parents, grandparents, etc. are. As a family tree is built back through generations, a most recent common ancestor (MRCA) is searched for. This is an ancestor that both the unknown and the DNA match share and have both descended from. Building the family tree forwards in time from the MRCA(s) and filling in multiple branches of the tree to generate family networks, could lead to a potential identity of the unknown, or narrow it down to a group of brothers or sisters within a family. Forensic DNA (STR) analysis is then used to confirm or refute the potential candidate as the identity of the unknown biological sample. The entire FGG investigation can be a complicated process as there are several forensic, genetic, and genealogical complexities that can be encountered throughout.

## 3. Consumer DNA Testing and Genealogy

### 3.1. Direct-TO-Consumer (DTC) DNA Testing

Consumer DNA testing, known as Direct-To-Consumer (DTC) DNA testing, has become a popular consumer product for individuals who are curious about their DNA, their biogeographic ancestry, and for some, the search for new or unknown genetic relatives. The first DTC DNA test, Oxford Ancestors (Bicester, UK), was released in 2000, and has since ceased trading. This was followed closely in the same month/year by FamilyTreeDNA, then 23andMe (South San Francisco, CA, USA) in 2007, AncestryDNA (Lee Hay, UT, USA) in 2012, and MyHeritage DNA (Oryehuda, Israel) in 2016. There have been many other DTC DNA tests (>30) released from other companies over the years, however, the four previously listed are considered the major DTC DNA testing companies as they are the most popular among consumers and therefore have the largest databases. Advances in technology, increased competition, and increased demand have not only driven down the price of DTC DNA tests (currently ~US$ 59–99) over the years but have also caused their respective databases to increase exponentially. The number of users in these databases (as of 16 July 2022) is estimated at; ~21 million in AncestryDNA, ~12.8 million in 23andMe, ~6 million in MyHeritage DNA, and ~1.77 million in FamilyTreeDNA [9]. This totals over 41 million people worldwide, in just the top four DTC DNA databases.

When a consumer purchases a DTC DNA test, they are provided with a sample collection kit (saliva sample or a cheek (buccal) swab), which the consumer then self-samples/collects and mails back to the company. The DTC testing company extracts the DNA from the sample and genotypes a large number of SNPs across the genome simultaneously using high-density SNP microarrays (‘SNP Chips’), e.g., the Illumina Infinium Global Screening Array (GSA). The type of genotyping microarrays used by DTC DNA companies can change over time. The Illumina Infinium GSA is currently the most widely used and may be customized for individual DTC DNA companies. A typical SNP array may analyze/genotype between ~600,000 and 1 million autosomal SNPs, and smaller numbers of Y chromosome, X chromosome, and mitochondrial DNA SNPs. Arrays are capped, for cost-effectiveness, at 1 million SNPs, with most companies having ~650,000 SNPs, which represents less than 1% of all the validated human polymorphisms in the dbSNP database. Genotyping arrays can harvest a lot of data at a relatively low cost and are therefore the popular choice for DTC DNA testing companies.

**Figure 1:**
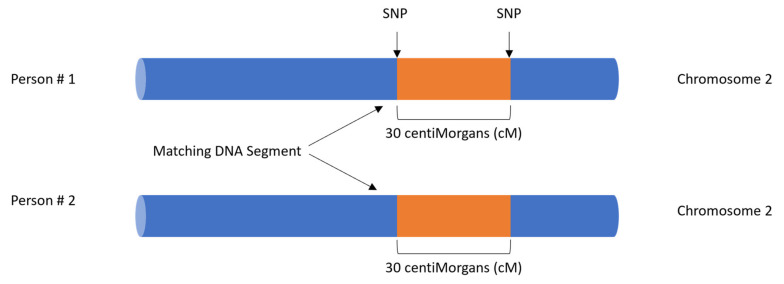
Matching DNA segment measured in centiMorgans (cM) for DNA matches.

When a consumer receives their DTC DNA test results, typically via app notification or email, the user can peruse their biogeographic ancestry predictions and/or search through a list of DNA matches in the database with whom they share DNA and are genetically related. These DNA relatives are identified by proprietary algorithms that search the genotyped data of each person, revealing matching DNA segments of sufficient size and cumulative amount to be able to infer the number of generations back to the most recent common ancestor between the two individuals and to predict their genetic relationship. The method of analysis to deduce this genetic relatedness is by determining if segments of DNA are shared identical by descent (IBD), meaning each individual has inherited the matching segment from a common ancestor without any intervening recombination. IBD segments of DNA are identified if all the alleles/genotypes in the segment on a chromosome are identical. The frequency of a segment must also be taken into consideration, as higher frequency matching segments in a population may indicate more distant relatives, while lower frequency matching segments may indicate relatives with common ancestry in a more recent generation. DNA matches may share half-identical regions (HIRs), meaning the matching DNA is either on the maternal or the paternal chromosome. Alternatively, DNA matches may share fully identical regions (FIRs), meaning the matching DNA is on both the maternal and the paternal chromosomes. This is sometimes observed in full siblings and double cousins. Endogamous populations may also display FIRs in distant relatives. The length of a matching segment is measured in centiMorgans (cMs), a unit of genetic distance, see Figure 1.

The cMs for all the IBD segments that meet a certain threshold are added to a total shared cM value. If a threshold for segment size is set too low it could cause false matches (i.e., unrelated individuals), while if a threshold is set too high it could exclude true matches (i.e., related individuals). The generally accepted minimum threshold for valid segments is 7 cM [10], as false positives have been observed with <7 cM segments. A study by Durand et al. [11] identified false positives in >67% of 2–4 cM segments.

It is important to note that no user is able to see the actual DNA data, i.e., the genotypes, for any other user in the database. However, each consumer can download their own raw DNA data, listing their genotypes for each SNP and the position on the genome. All uploaded data is tokenized within the database and it is the amount of DNA shared (total cM) between two individuals that is reported. Several DTC DNA databases may also report individual segment sizes, the chromosomes they are located on, the number of shared segments, and the longest shared segment. The total shared cM value (sometimes reported as a %) and the number of matching segments are used to predict the likely relationship. Typically, the larger the total shared cM value, the more closely related two people are. Precise relationship probabilities are not always possible because the ranges for a minimum and maximum of expected shared cMs and the number of segments for a particular relationship type will have overlap with other relationship types. For distant relationships, a relationship range is typically reported.

### 3.2. Predicting DNA Relationships

**Figure 2:**
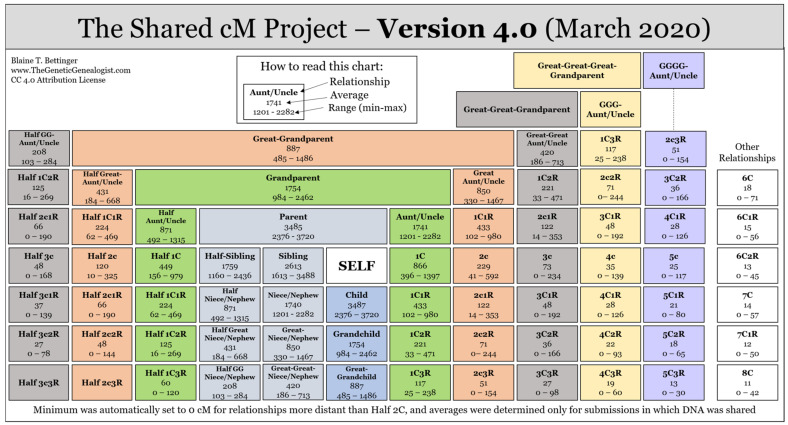
Shared cM Relationship Chart with the average and range (min-max) shared cMs for each relationship type. Image credit—Blaine T. Bettinger [14].

DTC DNA testing has become a popular tool for individuals searching for unknown genetic relatives. This can include adoptees searching for birth parents or vice versa, donor-conceived individuals searching for biological parentage, or other unknown parentage and/or kinship investigations. In addition, both hobbyists and professional genealogists have added DNA evidence from DTC DNA tests to their toolkit to confirm, refute, and even uncover new relationships in family trees. A number of factors however, can complicate genetic genealogy research, for example, DNA matches that are adopted, or have misattributed parentage, DNA matches who may have endogamy in their ancestral lines, or individuals whose top DNA matches have very low amounts of total shared DNA and are therefore very distant relatives, to name just a few. Within genetic genealogy databases (DTC DNA databases and/or GEDmatch), the predicted relationships based on the amount of shared DNA between two individuals should be interpreted with caution. For example, the average total cM for an aunt/uncle relationship is ~1740 cM, which is the same for a niece/nephew relationship. Therefore, supplemental information such as the persons age may aid in distinguishing which relationship is more likely. Additionally, the average total cM for a second cousin relationship is ~229 cM, however, can be as little as ~41 cM or as much as ~592 cM. Within this range, there is overlap with many other relationship types, e.g., first cousin (including once/twice/thrice removed), great aunt/uncle, third cousins, etc. Blaine Bettinger, a well-known genetic genealogist, maintains the Shared cM Project (ScP), a collaborative data collection project that collates the ranges of shared cMs from reported known relationships provided by genealogists across the world, with currently over 60,000 submissions [12,13]. A relationship chart, Figure 2 [14], is publicly available and regularly updated, that provides the ranges and average cMs for relationships, and has become an invaluable tool to genealogists worldwide.

In addition, DNA Painter, a third-party tool website, created by Johnny Perl, has the Shared cM Project 4.0 Tool [15], which incorporates data from the ScP, where users can enter a cM value and statistical probabilities of possible relationships will be generated, see Table 2 for the relationship probabilities for a DNA match with 229 cMs.

Therefore, a DTC DNA database/GEDmatch may predict a possible relationship type, however, there can be several relationship probabilities/hypotheses that need to be considered to identify the true relationship and position of that individual within a family tree.

In a 2018 study by Erlich et al. [16], the authors estimated that 60% of searches in genetic genealogy databases (with a dataset of 1.28 million people in this study) would likely identify DNA matches at the third cousin level or closer, with 15% at second cousin or closer. The authors indicate that a database containing only 2% of a target population would result in a third cousin match for 99% of the population. Similar predictions were reported by Edge et al. [17], who also provide an analysis of various theoretical models. The demographics of individuals within DTC DNA testing databases are suggested to have an over-representation of US citizens of European ancestry. As a result, this population may have greater chances of finding higher numbers of DNA matches/relatives, and possibly of a closer degree of relationship.

A limitation of searching DTC DNA databases for new/unknown relatives is that a person is limited to only finding DNA matches within the database of the particular company they have tested with. Many individuals may take DTC DNA tests from multiple companies or upload their DNA data from one testing company to another company that allows the upload of data. Both AncestryDNA and 23andMe do not allow the upload of DNA data from other companies, however, they both allow users to download their DNA data from their sites. FamilyTreeDNA and MyHeritage DNA allow users to both download their own DNA data and also upload their DNA data to their sites. Uploading DNA data, or testing with multiple companies, allows an individual to have a broader search, potentially comparing their DNA data to over 40 million people worldwide.

### 3.3. GEDmatch

Recognizing the need for establishing a centralized site/database where individuals could upload their DNA data from any DTC DNA company, Curtis Rogers (a hobbyist genealogist and retired business entrepreneur) and John Olsen (a retired engineer), founded GEDmatch in 2010. Their vision was to create a collaborative site where individuals could expand their genetic genealogy research by automating and mapping family trees, comparing their DNA data to a broad range of testers from different companies, and to develop a dashboard of tools to assist with interpreting the results. It is important to note, users have to upload their own DNA data manually to GEDmatch, and it is not a site that automatically pulls or imports DNA data from other DTC DNA databases. Anyone can create a free account on GEDmatch and can upload their own DNA data for comparison to other users. In 2014, the founders implemented advanced interpretation tools, known as Tier One tools, with a modest subscription fee. This fee for Tier One users was implemented in an effort to offset the costs of maintaining the site and the required servers, estimated to cost ~$200,000 per year at the time. GEDmatch is now a popular resource for individuals searching to expand their genetic genealogy research to uncover new genetic relatives. The suite of genetic genealogy tools in GEDmatch is unparalleled when compared to the somewhat limited tools available in DTC DNA databases.

In April 2018, when it was realized that GEDmatch had been used by law enforcement agencies for criminal investigation purposes, the founders of GEDmatch were shocked to learn of their database being used for this purpose, and many individuals/users criticized the ethical and privacy implications. At the time, the law enforcement use of GEDmatch did not breach any terms of service on the site. In May 2018, GEDmatch updated its terms of service to allow law enforcement uploads for the purpose of identifying perpetrators of violent crimes and identifying the remains of deceased individuals. The question of consent from the general users, however, stirred much discussion and controversy. In May 2019, the founders established an opt-in/opt-out system for users to decide for themselves if they wanted their DNA data/kit(s) to be compared to law enforcement uploads. All kits that were already in GEDmatch were automatically set to opt-out, and users had to manually change their settings to opt-in for law enforcement matching if they wished to. New users/uploads to the site, were presented with the opt-in/opt-out function during the new upload process.

As the use of FGG continued to grow, and indeed the number of users in GEDmatch continued to grow, the founders recognized they required additional resources beyond those they had and their small group of volunteer programmers. In addition, the regulation of the law enforcement use of the site and the complex issues of privacy from a legal and ethical perspective, extended beyond the founder’s areas of expertise. In May 2019, it was announced that GEDmatch had been purchased by Verogen, a forensic genomics company, formed in 2017 as a spin-off entity of Illumina (San Diego, CA, USA), a well-known DNA sequencing company. Curtis Rogers released a letter to all GEDmatch users affirming that Verogen’s acquisition of GEDmatch was a positive step forward for the benefit of the broader genetic genealogy community as Verogen has the resources needed to enhance the site and to develop systems to oversee law enforcement use and privacy. Since Verogen’s acquisition of GEDmatch many updates and changes have been implemented to the site. A new updated interface with improved user functionality was established early on. Some existing tools have been improved and new tools have been added to enhance the user’s ability to extrapolate information from DNA matches about their relationships and family tree connections.

In December 2021, the terms of service in GEDmatch for general users, related to the opt-in/opt-out system for law enforcement matching for general users, was updated to reflect changes that had been previously made in January of that year to the opt-in/opt-out definitions. Opting-in remained the same, i.e., users are compared to all law enforcement uploads for violent crimes and unidentified human remains. Opting-out, however, changed to only being excluded from comparisons of law enforcement uploads for violent crimes, with all users now included in comparisons for unidentified human remains.

The most recent update to GEDmatch, as of 1 May 2022, allows users with free accounts to upload up to five DNA data files, called “kits”, and have access to several free interpretation tools. Free users previously had no limit on the number of kits they could upload. Users who want to upload more than five DNA data files/kits and have access to more advanced interpretation tools can pay a subscription for a Tier One account. Currently, Tier One subscription costs $15 per month on a pay-as-you-go basis, or $10 per month on a recurring basis, or $100 per year. The number of users/kits in GEDmatch is currently reported to be ~1.8 million, with >500,000 opted-in for law enforcement comparisons, and an estimated 75% of new users choosing to opt-in (sourced from personal communication with Verogen).

### 3.4. Genealogical Research

Identifying genetic relatives in DTC DNA databases and/or GEDmatch is just one part of the process for individuals seeking to identify biological parentage, or to build out their extended family tree(s). While the DNA databases are large, not every person in the world has tested. Therefore, to build family trees from DNA matches, genealogical research is required to identify the other members of a family tree. The genealogical research required to accurately place that individual into the correct position within a family tree can be very complex and requires expertise in genealogical research to collect and correlate accurate documentary evidence.

In 2014, Time Magazine stated that Genealogy is the second most popular hobby in the world, after gardening, and is the second most searched category on the internet [18]. Indeed, there are several professional organizations across the world that are dedicated to Genealogy, and also several university certificate and degree programs that focus on genealogy and family history research. The Association of Professional Genealogists (APG), a not-for-profit 501(c)(6) organization, lists ~3000 members in forty countries around the world. While genealogy may be a hobby or a past-time for some, professional genealogy is a discipline/field of study that requires expertise, developed through training, education, and experience. The Board for Certification of Genealogists (BCG), a US-based non-profit organization founded in 1964, is a professional credentialing body for genealogists. The BCG sets standards for competence and ethics in genealogical research through the publication of the Genealogy Standards [19]. The Genealogy Standards sets 90 standards addressing; documentation, research planning and execution, including reasoning from evidence, compiling research results, genealogical education, and ongoing development of genealogical knowledge and skills. These standards seek to ensure sound genealogical research and accurate research outcomes. In October 2018, the BCG updated the standards in response to the increase in consumer DNA testing and this new type of DNA evidence added to the genealogist’s toolkit. Four existing standards were modified and seven new standards were added to guide the use of DNA evidence in genealogical research [19,20]. The Genealogical Proof Standard (GPS) is used to measure the credibility of conclusions reached when identifying ancestral identities, relationships, and events, that overarch all of the genealogy standards for documenting, research, and writing. The five components of the GPS include;

1. Reasonably exhaustive research.
2. Complete and accurate source citations.
3. Critical tests of relevant evidence through processes of analysis and correlation.
4. Resolution of conflicting evidence.
5. Soundly reasoned, coherently written conclusion.

Genealogical research uses historical records and other sources to build family trees and verify identities and kinship. Information derived from quality sources (e.g., original records) and the evidence of events/relationships that can be drawn from them are key to reaching reliable conclusions, with collected documentation supporting those conclusions. There are a variety of record collections that are used in genealogical research, such as vital records, also known as civil registration records, e.g., birth, death, marriage records. Other records include adoption records, census records, church and religious records, city directories, court records, emigration, immigration, and naturalization records, land and property records, military records, newspaper articles, obituaries, cemetery records, tax records and voter records, to name just a few. Genealogical research can be a time-consuming and laborious task and may require visiting city/state/national archives to locate original records. In recent years, however, a large number of records have been digitized and are readily accessible online, such as on the U.S. National Archives, Ancestry, Newspapers, FamilySearch, and Fold3 websites, plus more, with some requiring subscriptions for a fee. Various factors can cause sourcing accurate documentary evidence difficult, for example, a lack of records or destroyed records, locating international records, translating languages in records, a lack of understanding of naming conventions for certain populations, name changes or misspellings on immigration records at ports of entry, to name just a few. During genealogical research, when individuals and relationships are identified they are entered into a family tree with their associated data. There are a number of family tree building platforms/software programs, e.g., RootsMagic, Family Tree Builder, or the family tree platform within Ancestry. Some genealogists prefer other methods, e.g., paper-based, Microsoft Excel, or LucidChart.

Ethics in genealogical research is imperative, for the protection of the public, the client, and the profession. Protecting the privacy of individuals, in particular living individuals, is necessary, and the possibility of revealing family secrets or unwanted information is always possible. When genealogical research is performed by experienced, educated, and ethical researchers, credible conclusions can be reached to provide a comprehensive analysis of an individual’s family lineage.

## 4. Forensic Genetic Genealogy in Practice

### 4.1. Case Types

Based on data from the FBI’s Uniform Crime Reports (UCR), experts estimate there are greater than 240,000 unresolved homicides in the US, which increases by approximately 6000 each year [21,22]. It is estimated that less than half of the unsolved homicides have DNA evidence available. In 2007, the journal of the National Institute of Justice (NIJ) stated that Missing Persons and Unidentified Persons are the United States’ silent mass disasters [23]. The National Missing and Unidentified Persons System (NamUs), a national information clearinghouse and resource center for missing, unidentified, and unclaimed person cases in the US currently lists >21,700 open missing person cases and >14,000 open unidentified persons cases [24]. The application of FGG to these large numbers of unresolved violent crimes and UHR cases could greatly contribute to clearing these cases. The term “cold cases” originated in the media and has become a more popularized term for unresolved cases. Cases are unresolved when all investigative leads are exhausted, yet the case remains open while awaiting new evidence to come to light. While FGG has been successful in resolving many cold cases, some decades old, FGG can also be applied to more recent unresolved cases, providing forensic DNA profiling/CODIS search has already been performed and resulted in no probative leads. The use of FGG in recent unresolved cases could greatly benefit public safety if a perpetrator of a violent crime was identified and apprehended in a shortened timeframe, thereby reducing the risk of them re-offending or harming anyone else.

In a study by Dowdeswell [25], it was reported a total of 439 cases had been resolved where FGG had provided an investigative lead, prior to 31 December 2020. The author reached this total case number by collecting all publicly available information on cases where FGG had been cited as being used in the case, e.g., internet searches, discussion boards, press releases, publications, and published court records. It was noted that not all cases would be identified using these methods, as not all cases are publicly released. This study also performed a systematic review of the cases on a number of metrics, e.g., case type, victim demographics, perpetrator demographics, etc. The results highlighted that the majority of FGG cases involved serial/recidivist offenders and sexual violence. Victims are predominantly female and from low-income, vulnerable social groups, while perpetrators are predominantly male, young, and of European ancestry [25].

### 4.2. FGG Databases

Currently, there are three genetic genealogy databases that explicitly allow the upload of SNP data files from forensic samples for FGG purposes (i.e., law enforcement kits). These are; GEDmatch PRO, FamilyTreeDNA, and DNASolves.

Verogen launched GEDmatch PRO in December 2020 to create a separate and dedicated portal for law enforcement to upload SNP data files to for FGG purposes, and to perform the genetic genealogy analyses within. The GEDmatch PRO portal contains many of the useful genetic genealogy tools found in regular GEDmatch. Once GEDmatch PRO was launched, the terms of service in GEDmatch were updated to reflect that all law enforcement kits must now only be uploaded through GEDmatch PRO. Agencies and FGG experts must agree to the terms of use for GEDmatch PRO, prior to creating an account and uploading a kit. The terms of use require that law enforcement may only use the service to identify the perpetrator of a violent crime or to identify human remains, and only in a manner that complies with all laws. In April 2022 the terms of use were updated to clarify that unidentified human remains cases exclude fetal remains and the remains of stillborn children. Investigators must create an account within GEDmatch PRO, using a professional email address and contact information, and select from one of three roles; law enforcement, forensic lab, or genealogist. Once a law enforcement kit has been uploaded and finished batch processing, the kit will appear in the dashboard. Each kit can be assigned to a project where individual investigators/genealogists can be added to the project to gain access to the kit. All law enforcement kits that were uploaded to regular GEDmatch prior to the release of the PRO portal were transferred over.

FamilyTreeDNA (FTDNA), a popular DTC DNA testing company, changed their terms of service in December 2018 to allow law enforcement to use their site for FGG purposes. FTDNA publishes a Law Enforcement Guide on their website and specify that permission is only granted in cases to identify a perpetrator of homicide, sexual assault, or abduction, and to identify the remains of a deceased individual [26]. FTDNA grants permission to use the service after the required documentation is submitted, reviewed, and approved, and prior to uploading to the database. The FTDNA database has greater than 1.7 million user datafiles. All general users (i.e., non-law enforcement) can opt-in or opt-out for law enforcement matching, with an estimated 96% of US users (data sourced in 2020) within the database opted-in [27]. FTDNA has a suite of genetic genealogy tools available within their database, e.g., a chromosome browser, chromosome painting, biogeographic ancestry prediction, matrices, etc. FTDNA also has a sister sequencing company, Gene-By-Gene, which offers SNP sequencing services to law enforcement agencies, which includes automatic upload to the FTDNA database.

DNASolves was launched in November 2019 by forensic genomics company Othram, Inc. This database was intentionally designed for FGG purposes only. Willing individuals can upload their own raw DNA data if they have previously taken a DTC DNA test, or they can request a DNA collection kit from Othram, for inclusion in the DNASolves database. The difference with this database is that individuals who volunteer/upload their DNA data are doing so solely to aid in FGG investigations and they will not have access to the database for genealogical research or to view their own DNA matches within the database. The terms of use make this clear to all individuals volunteering their DNA data to the database. Individuals are asked to provide as much family tree data as possible, or to upload a GEDcom file (a genealogical data file). Law enforcement agencies may contract the services of Othram for SNP sequencing of forensic DNA samples and FGG analyses, including searching the DNASolves database.

It is important to note that many of the principal DTC DNA testing companies changed their terms of service prohibiting law enforcement’s use of their databases for FGG purposes, e.g., AncestryDNA, MyHeritageDNA, and LivingDNA. Their specific policies and terms of service can be located on each of their websites.

### 4.3. The FGG Workflow

As the majority of case investigations utilizing FGG to date have occurred predominantly in the US, the following workflow is based on typical and recommended processes employed in the US. Other countries may have currently, or in the future, differing case criteria, methodologies, and policies.

#### 4.3.1. Case Assessment/Criteria

Within the US, per the US Department of Justice (DOJ) interim policy on the use of FGG [28], FGG may be pursued in cases of unsolved violent crimes (i.e., homicide and sexual assault) where the forensic sample is believed to be from the putative perpetrator, or cases of unidentified human remains (UHR cases). In addition, FGG may be authorized in other violent crimes or attempts to commit violent crimes that present a substantial and ongoing threat to public safety and national security.

Some law enforcement agencies in the US have developed their own in-house FGG units (non-laboratory based), while some have their own trained FGG investigators and may additionally contract the services of an external FGG consultant/expert. Other agencies outsource cases in their entirety to private FGG providers. Investigative collaboration and communication between the law enforcement agency, the forensic laboratory, and the FGG provider(s) is necessary to reserve the integrity of an investigation and also to ensure the chain of custody of the evidence sample(s) is maintained. Biological samples are typically in the possession of the forensic laboratory and/or Medical Examiners Office and therefore they must be consulted (e.g., a Designated Lab Official (DLO) in state forensic laboratories in the US) to determine the current status and eligibility of the forensic sample. As per the US DOJ interim policy for the use of FGG a case must have first generated a forensic DNA/STR profile and have failed to identify a candidate match in CODIS, prior to pursuing FGG. A quality and quantity assessment of the remaining biological evidence in a case is an essential step in the FGG workflow. If an original DNA extract of the forensic sample is remaining, the extract volume, quantity data, and quality data must be reviewed. If an original DNA extract of the forensic sample is not remaining, a review of the biological sample (e.g., blood, semen, tissue sample) for suitability of re-extraction is performed. Qualitative and quantitative data of the forensic sample is key to informing the type of SNP analysis technology to be employed.

#### 4.3.2. SNP Analysis

SNP microarrays are the most cost-effective method to perform (~US$600–800 per sample) to generate the SNP data for genetic genealogy analysis purposes, however they require high-quantity and high-quality samples. Approximately 200 ng of genomic DNA, with a good DNA Degradation Index (DI) score, are recommended for SNP microarrays, although some have reported success with lower quantity samples. As forensic samples are often compromised/degraded from exposure to various environmental insults, and can be much reduced in quantity and quality, a more comprehensive SNP analysis technology may be required to generate sufficient SNP data for genetic genealogy purposes. Whole Genome Sequencing (WGS) is a favored method for compromised samples, albeit a more expensive method to perform (~US$1000–$2000 per sample). WGS can provide autosomal coverage of 30X to 100X, thereby potentially producing sufficient data that a SNP microarray may not be able to achieve with compromised/degraded samples. The recommended input of genomic DNA is ~100 ng for WGS, however successful analysis has been achieved with less [1,29]. A third SNP analysis technology has recently been introduced and designed specifically for FGG applications. A targeted SNP kit was developed by Verogen and was commercially launched in early 2021. The ForenSeq^®^ Kintelligence Kit targets 10,230 forensically curated SNPs and can be analyzed using Illumina’s MiSeq FGx sequencing platform. The curation of the SNPs was aimed to select those that provide the most probative information for genetic relatedness, have overlap with the common microarrays (e.g., the Illumina Infinium GSA) for matching/comparison purposes within the databases, and importantly, to eliminate/minimize the analysis of SNPs that are medically informative. The recommended input of DNA is 1 ng for the Kintelligence kit, with demonstrated performance as low as 100 pg, reported by Verogen. It is important to note that forensic laboratories in the US (state/federal) do not have the required infrastructure/instrumentation to perform SNP microarrays or WGS in-house. Therefore, samples must be outsourced to private vendor laboratories for sequencing. As Next Generation Sequencing (NGS) platforms can provide analysis of several key interest areas in the forensic genetics field, some forensic laboratories have begun implementing NGS platforms into their laboratory workflow, both in the US and internationally. Therefore, there is potential in the future for forensic laboratories that have implemented NGS in their laboratory to be able to run the Kintelligence kit on samples in-house.

#### 4.3.3. Genetic Genealogy Database Upload and Analysis

Once the sequencing of a sample is complete, and a FASTQ file is generated, an assessment of the data is first performed by bioinformatics analysis, and genotype imputation and phasing may be required, particularly for data from degraded DNA samples. A detailed discussion on SNP genotype imputation is provided in a review paper by Kling et al. [30]. The resulting SNP data file is then uploaded to one of the law enforcement-approved genetic genealogy databases, following communication and approval from the database stewards. When the datafile (i.e., “the Kit”) has finished batch processing, a kit number will be assigned, and the genetic genealogy analysis can begin. Individual FGG experts/providers follow their own genetic genealogy workflow using the various tools within the database(s) and third-party tools. Detailed discussion of the various genetic genealogy tools used in FGG investigations is outside the scope of this paper; however, there are several key tools that can prove very fruitful for harvesting valuable information. This can include an assessment of the biogeographic ancestry of the unknown, how DNA matches not only match the unknown on a one-to-one basis, but also how the matches match each other, grouping them in to a set based on common ancestor(s), and more. As the field of genetic genealogy continually evolves, new tools are regularly introduced that further enhance the interpretation of the available data. It is therefore necessary that FGG investigators continually update their knowledge and education of the available tools. The suite of tools, and the knowledge of how to use them and how to interpret their results, is key to extrapolating as much information as possible to deduce the genetic connections. Without this knowledge, a case could become compromised and deemed “unsolvable” as key probative information could be missed/overlooked, as well as wasting valuable time and resources.

#### 4.3.4. Genealogical Research

Once the genetic genealogy analysis has been thoroughly performed, DNA matches are selected for further genealogical research. The identities of the DNA matches should be confirmed using accurate, collected records, and comprehensive family trees are constructed of extended family members and ancestral lines are traced. Records/documentary evidence, with appropriate citations, that prove key relationships/kinships and events (e.g., marriages/births/deaths) should be collected and stored. In FGG investigations, the family tree(s) should be constructed in secure platforms that present no privacy/confidentiality risks. The tree(s) should not be publicly accessible/viewable. There are currently no standards in place specifically for FGG investigations; however, it is the author’s opinion that the Genealogy Standards and the Genealogical Proof Standard of the BCG are a good template to work from and aim for. It is important to note that all evidence/data/records/analyses/family trees collected or built throughout an FGG investigation should be treated as forensic evidence and could potentially be requested during the discovery process in court proceedings, and therefore should be readily available.

#### 4.3.5. Potential Candidate Identification

The genealogical research may lead to a possible candidate or candidates for the identity of the unknown. Multiple candidates may be suggested in cases where the genealogical research leads to a family of multiple brothers or multiple sisters. The genealogical research cannot determine which one of the brothers/sisters the unknown is suggested to be, however biographic information such as age, known addresses at the time of the crime, etc. may assist in narrowing it down to one brother/sister. In some cases, the genealogical research may reach a point where a living individual, or multiple individuals, in a family tree are identified and who are believed to be closely related to the unknown (i.e., potentially first or second cousins). A process commonly known as target testing can be performed, whereby a DNA sample/data is requested from these individuals (“target testers”). Written informed consent should be obtained from target testers in all cases. If the individual has previously taken a consumer DNA test, they may be requested to upload their data to GEDmatch/FTDNA and to opt-in for law enforcement matching. Alternatively, they may be provided with a consumer DNA test or they may have a reference DNA swab collected and SNP sequenced, then uploaded. Genetic genealogy comparisons between the target tester(s) and the unknown are performed. This may potentially narrow the genealogical research to particular branches of a family tree and assist in leading to a possible identity of the unknown.

#### 4.3.6. STR Profile Confirmation

Once a possible identity of the unknown has been reached, as per the US DOJ interim policy [28], a reference DNA sample of the potential candidate must be collected, forensic STR profiled, and compared to the original forensic profile, prior to any arrests being made. This process falls outside the scope of outsourced FGG providers and is the responsibility of the investigating law enforcement agency. Reference DNA samples are collected by the law enforcement agency by requesting a sample from the candidate, or through surreptitious DNA collection, subject to all legal requirements for such collection. The resulting reference STR profile comparison to the forensic unknown will either confirm or refute the findings of the FGG investigation.

## 5. Current and Future Considerations

When FGG emerged in criminal investigations in the US in 2018, there were no restrictions, guidelines, or policies on its use at first. In an effort to place some oversight on its use, the US Department of Justice (US DOJ) published the first interim policy on the use of FGG, which went into effect in November 2019 [28]. The interim policy contains nine sections that outline the critical requirements for the use of FGG by law enforcement, including the criteria a case must meet for the use of FGG. The interim policy is designed to ensure that FGG is used responsibly to solve violent crimes and to balance protecting public safety with the privacy and civil liberties of all citizens. A final policy was projected for release in 2020, which is still yet to be released [31]. The state of Maryland was the first US state to enact its own law regulating the use of consumer genetic data by law enforcement [32]. Legislatures convened a working group to gain insight and perspectives from representatives from key areas, including forensic, genetic genealogy, legal, ethical, and criminal justice professionals and experts. The resulting legislation has been suggested as a model for others moving forward [33]. Some key features of the Maryland law are it requires; judicial authorization to initiate the use of FGG, consent is obtained from users in databases for law enforcement matching, informed consent is obtained from target testers and prohibits covert collection, FGG is available for criminal defendants seeking to prove guilt or innocence, there are consequences for the misuse of FGG and its associated data, and lastly, annual reporting of the use of FGG. Other states are following suit and have begun to propose and/or enact their own state specific laws, for example, Montana’s law [34] that focuses on search warrants, and Utah’s proposed bill [35] which failed. It is expected that other states will also consider drafting their own legislation for the regulation of FGG by law enforcement, which is welcomed by many within the FGG community, including this author, to ensure the responsible, legal, and ethical use of this tool.

As DNA sequencing technologies continue to advance, it is important that the genetic privacy of individuals is considered, and ethical boundaries are not crossed. DTC DNA testing companies have a responsibility to protect the privacy of their consumers, and similarly, those consumers of DTC DNA tests have a personal responsibility to be informed of what they are agreeing to. This also includes consumers uploading their data to genetic genealogy databases such as GEDmatch. Ethical and privacy considerations on the overall practice of FGG are discussed in broad forums (e.g., industry meetings/conferences, institutional webinars, forensic committees/working groups, etc.) and there are published articles on this topic that provide valuable insight [36,37]. The ethics and privacy considerations should continually be discussed and reviewed as the field continues to grow to ensure the best interests of all citizens are protected.

Prior to 2018, there did not exist any law enforcement agencies or private companies offering FGG services for hire. Today, many FGG units have been formed within various state and federal law enforcement agencies in the US, with the Florida Department of Law Enforcement (FDLE) establishing the first state-level in-house FGG unit, followed by the Federal Bureau of Investigation (FBI) establishing their own unit also. In addition, there are now numerous private companies/individuals offering FGG services to law enforcement agencies, typically on a case-by-case contract basis. Some of these companies have a long history as providers of forensic services to law enforcement agencies, for example, Bode Technology, and have added FGG to their suite of services offered. Many new companies however have now emerged in the last four years that offer FGG services, with some providing the laboratory analysis for the generation of the SNP data, some providing only the genetic genealogy analysis/research, and some providing both, i.e., full FGG service. It is recommended that contracts with external vendor laboratories/providers of FGG service be reviewed by legal counsel to ensure appropriate privacy and security controls are in place for the handling of forensic evidence/biological samples, genetic data, and other collected information pertaining to a case. Quality Systems (quality assurance and quality control) are a key feature of forensic laboratories, and it is essential that external vendor laboratories maintain similar systems, to protect the credibility of analyses performed within them.

As stated previously, there are currently no specific FGG standards or best practices established, which are much needed, to address the many individual components and intricacies within an FGG investigation. Individual FGG providers/experts can essentially set their own standards and best practices for carrying out an FGG investigation, and some may operate on an ad hoc basis. It is the authors belief that the development of FGG standards is an emergent need that is critical to ensuring the integrity and sustainability of this field. FGG standards should address many key components of FGG, for example standards for; case management, processing and handling of genetic evidence/data, outsourcing to vendor labs/providers, documentary evidence and tree building, and reporting/written testimony of FGG analyses and conclusions, plus more. Building upon and combining standards that already exist independently in the fields of genealogy, forensic DNA, criminal justice, and the judicial system will ultimately strengthen the resulting standards. To draft these standards, input is needed from all stakeholders, including forensic practitioners/scientists, genealogy professionals, legal professionals, law enforcement professionals, ethics experts, and privacy experts, plus more.

## 6. Conclusions

Forensic DNA profiling revolutionized the fields of forensic science and criminal investigations when it first emerged in the mid-late 1980s, followed by it’s widespread implementation in the 1990s. As technology and knowledge advance, new tools come to the forefront, to not only enhance, but also complement our existing methods. FGG combines the use of forensic DNA evidence, SNP testing, genetic genealogy databases, and genealogical research for the purposes of human identification. While FGG is still in its infancy, the field has grown tremendously since 2018. The scientific and technical aspects of FGG have been developing rapidly, including but not limited to, the implementation of additional SNP analysis technologies and the ongoing research, development, and release of new SNP marker sets and kits purposefully designed for FGG purposes. Legal, ethical, and privacy considerations have been, and continue to be, explored, including ongoing efforts to regulate the use of FGG. 

While the impact FGG has had in resolving hundreds of cases in a few short years cannot be denied, to ensure its continued success, it is essential that it is used responsibly, ethically, and with sound scientific practices. In addition, appropriate forensic, investigative, and criminal procedure standards must be followed throughout, with the ultimate goal of protecting public safety and individual privacy at the forefront.

## Figures and Tables

**Table 1:** Summary of differences for Forensic DNA Analysis and Forensic Genetic Genealogy.

	Forensic DNA Profiling	Forensic Genetic Genealogy
**DNA markers**	Short Tandem Repeats (STRs)	Single Nucleotide Polymorphisms (SNPs)
**Region of Genome**	Non-coding region	Coding region
**Number of Markers**	16–27	>10,000 for targeted SNP kits, >600,000 for SNP microarrays
**Current Technology**	PCR Amplification and Capillary Electrophoresis	Next Generation Sequencing, Whole Genome Sequencing, Targeted SNP Kits
**Data File Generated**	Electropherogram	FASTQ
**Databases Searched**	National (criminal) DNA Databases	Genetic Genealogy Databases (approved for Law Enforcement Use)

**Table 2:** Predicted relationship probabilities with % confidence for a 229 cM match in DNA Painter’s shared cM Tool.

229 cM Match—Relationship Probabilities	
54%	Half GG-Aunt/Uncle, 2C, Half 1C1R, 1C2R, Half GG-Niece/Nephew
35%	Half 2C, 2C1R, Half 1C2R, 1C3R
9%	Great-Great-Aunt/Uncle, Half Great-Aunt/Uncle, Half 1C, 1C1R, Half Great-Niece/Nephew, Great-Great-Niece/Nephew
3%	Half 1C3R, Half 2C14, 3C, 2C2R

## Data Availability

Not Applicable.
